# Guava (*Psidium guajava* L.) Leaves: Nutritional Composition, Phytochemical Profile, and Health-Promoting Bioactivities

**DOI:** 10.3390/foods10040752

**Published:** 2021-04-01

**Authors:** Manoj Kumar, Maharishi Tomar, Ryszard Amarowicz, Vivek Saurabh, M. Sneha Nair, Chirag Maheshwari, Minnu Sasi, Uma Prajapati, Muzaffar Hasan, Surinder Singh, Sushil Changan, Rakesh Kumar Prajapat, Mukesh K. Berwal, Varsha Satankar

**Affiliations:** 1 Chemical and Biochemical Processing Division, ICAR—Central Institute for Research on Cotton Technology, Mumbai 400019, India; manoj.kumar13@icar.gov.in; 2 ICAR—Indian Grassland and Fodder Research Institute, Jhansi 284003, India; Maharishi.tomar@icar.gov.in; 3 Institute of Animal Reproduction and Food Research, Polish Academy of Sciences, Tuwima 10 Str., 10-748 Olsztyn, Poland; 4 Division of Food Science and Postharvest Technology, ICAR—Indian Agricultural Research Institute, New Delhi 110012, India; vivek_11593@iari.res.in (V.S.); uma_11103@iari.res.in (U.P.); 5 Department of Nutrition and Dietetics, Faculty of Allied Health Sciences, Manav Rachna International Institute of Research and Studies, Faridabad 121004, Haryana, India; sneha.fas@mriu.edu.in; 6 Department of Agriculture Energy and Power, ICAR—Central Institute of Agricultural Engineering, Bhopal 462038, India; Chirag.Maheshwari@icar.gov.in; 7 Division of Biochemistry, ICAR—Indian Agricultural Research Institute, New Delhi 110012, India; minnusasi1991@gmail.com; 8 Agro Produce Processing Division, ICAR—Central Institute of Agricultural Engineering, Bhopal 462038, India; Muzaffar.Hasan@icar.gov.in; 9 Dr. S.S. Bhatnagar University Institute of Chemical Engineering and Technology, Panjab University, Chandigarh 160014, India; ssbhinder@pu.ac.in; 10 Division of Crop Physiology, Biochemistry and Post-Harvest Technology, ICAR—Central Potato Research Institute, Shimla 171001, India; Sushil.Changan@icar.gov.in; 11 School of Agriculture, Suresh Gyan Vihar University, Jaipur 302017, Rajasthan, India; rakesh.prajapat@mygyanvihar.com; 12 Division of Crop improvement, ICAR—Central Institute for Arid Horticulture, Bikaner 334006, India; Mukesh.kumar4@icar.gov.in; 13 Ginning Training Centre, ICAR—Central Institute for Research on Cotton Technology, Nagpur 440023, India; satankarvarsha@gmail.com

**Keywords:** phenolic extracts, essential oils, polysaccharides, valorization, crop residue

## Abstract

*Psidium guajava* (L.) belongs to the Myrtaceae family and it is an important fruit in tropical areas like India, Indonesia, Pakistan, Bangladesh, and South America. The leaves of the guava plant have been studied for their health benefits which are attributed to their plethora of phytochemicals, such as quercetin, avicularin, apigenin, guaijaverin, kaempferol, hyperin, myricetin, gallic acid, catechin, epicatechin, chlorogenic acid, epigallocatechin gallate, and caffeic acid. Extracts from guava leaves (GLs) have been studied for their biological activities, including anticancer, antidiabetic, antioxidant, antidiarrheal, antimicrobial, lipid-lowering, and hepatoprotection activities. In the present review, we comprehensively present the nutritional profile and phytochemical profile of GLs. Further, various bioactivities of the GL extracts are also discussed critically. Considering the phytochemical profile and beneficial effects of GLs, they can potentially be used as an ingredient in the development of functional foods and pharmaceuticals. More detailed clinical trials need to be conducted to establish the efficacy of the GL extracts.

## 1. Introduction

Plants are a predominant natural source of numerous bioactive compounds [1,2]. Several diseases have been cured using a variety of plant preparations in folk medicine since ancient times [3] and, presently, cosmetic, pharmaceutical, and nutraceutical industries are paying more attention to plant preparations and pure phytochemicals. The projected growth of the plant preparation market is around USD 86.74 billion by 2022, with the largest market share belonging to the pharmaceutical sector, followed by the nutraceutical industry. Interestingly, the utilization of plant preparations for cosmetics, beverages, food, and medicine is mainly dependent on plant leaves. Among all plant organs, leaves are the largest accumulators of bioactive compounds, such as secondary metabolites. Several recent studies reported phytochemical profiles and biological activities of leaf extracts of various cultivated plants [2,4,5,6]. Hence, although plant leaves are considered as agricultural waste, they are a rich source of high-value nutra-pharmaceutical compounds.

**Figure 1:**
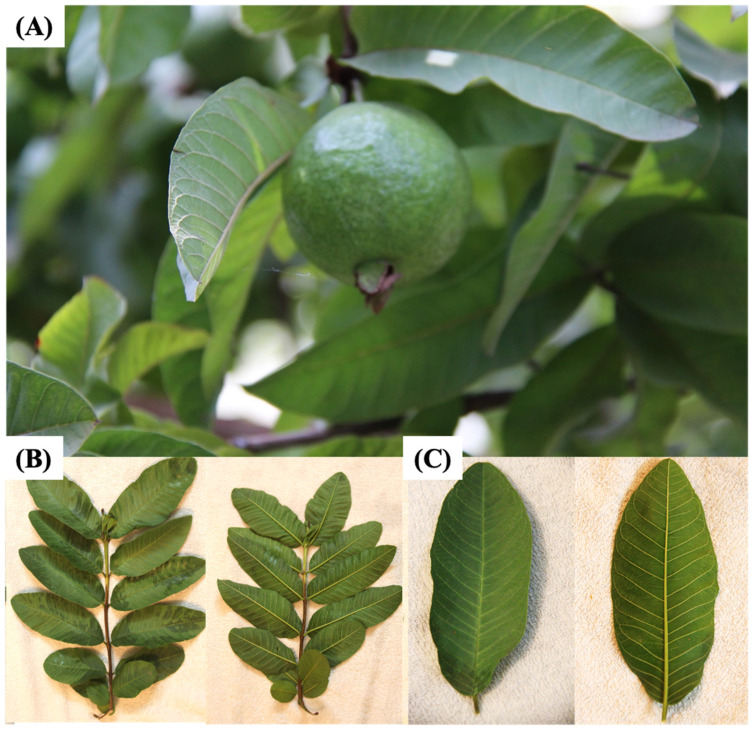
(**A**) Guava fruit and leaves, (**B**) bunch of guava leaves with dorsal view on the left and ventral view on the right, (**C**) guava leaf with dorsal view on the left and ventral view on the right.

The guava (*Psidium guajava* L.) tree (Figure 1), belonging to the Myrtaceae family, is a very unique and traditional plant which is grown due to its diverse medicinal and nutritive properties. Guava has been grown and utilized as an important fruit in tropical areas like India, Indonesia, Pakistan, Bangladesh, and South America. Different parts of the guava tree, i.e., roots, leaves, bark, stem, and fruits, have been employed for treating stomachache, diabetes, diarrhea, and other health ailments in many countries. Guava leaves (*Psidii guajavae folium*; GL) are dark green, elliptical, oval, and characterized by their obtuse-type apex. Guava leaves, along with the pulp and seeds, are used to treat certain respiratory and gastrointestinal disorders, and to increase platelets in patients suffering from dengue fever [7]. GLs are also widely used for their antispasmodic, cough sedative, anti-inflammatory, antidiarrheic, antihypertension, antiobesity, and antidiabetic properties [8]. Studies on animal models have also established the role of GL isolates as potent antitumor, anticancer, and cytotoxic agents [9,10].

GLs are widely employed for treating diarrhea and digestive ailments, while the fruit pulp is utilized to enhance the platelet count for treating dengue fever. The potential of guava leaf extracts for diarrhea treatment was also studied [11,12]. The flavonoids present in guava leaf extract chiefly determine their antibacterial activity, while quercetin, which is the most predominant flavonoid of guava leaves, exhibits strong antidiarrheal activities. The antidiarrheal activity of quercetin is ascribed to its relaxing effect on the intestinal muscle lining which prevents bowel contractions. Guava leaf polysaccharides (GLPs) can be utilized as an antioxidant additive in food and for diabetes treatment.

The presence of a unique variety of bioactive polyphenolic compounds, like quercetin and other flavonoids, and ferulic, caffeic, and gallic acids, present in guava leaves primarily determine their bioactive and therapeutic properties [8,13]. These phenolic compounds are known as secondary metabolites which exhibit strong antioxidant and immunostimulant activities. This review aims to discuss the various nutritional and bioactive compounds present in guava leaves and decipher the molecular basis of their pharmacological and medicinal properties concerning human health, nutrition, and as complementary medicine.

## 2. Chemical Composition

### 2.1. Proximate Composition

Guava leaves (GLs) are a rich source of various health-promoting micro- and macronutrients as well as bioactive compounds. They contain 82.47% moisture, 3.64% ash, 0.62% fat, 18.53% protein, 12.74% carbohydrates, 103 mg ascorbic acid, and 1717 mg gallic acid equivalents (GAE)/g total phenolic compounds [14]. The overall proximate profile of GLs is presented in Table 1.

#### 2.1.1. Polysaccharides

Polysaccharides are macromolecules that are ubiquitously present in nature. They are made of long polymeric chains, which are composed of monosaccharide units. These polysaccharides demonstrate various physicochemical, biological, and pharmacological properties, such as antioxidant, anti-inflammatory, antidiabetic, immunomodulatory, and antitumor activities [15]. Guava leaf polysaccharides (GLPs) can be isolated using ultrasound-assisted extraction (UAE) (time: 20 min, power: 404 W, temperature: 62 °C). These GLPs contain about 9.13% uronic acid and 64.42% total sugars, out of which 2.24% are reducing sugars. GLPs are soluble in water, while insoluble in organic solvents like ethanol, diethyl ether, ethyl acetate, acetone, and chloroform. Extracted GLP with a concentration of 100 μg/mL exhibits good antioxidant capacity with 56.38% and 51.73% 2,2-diphenyl-1-picrylhydrazyl (DPPH) radical- and 2,2′-azino-bis(3-ethylbenzothiazoline-6-sulfonic acid (ABTS) radical cation-scavenging capacity, respectively [16]. Similar results were also reported by Kong et al. [17]. They obtained up to 0.51% GLP using UAE that exhibited good DPPH^•^- and ^•^OH-scavenging activity (72–86% and 42.94–58.33%). GLPs can be categorized into two groups: unsulfated and sulfated GLPs. Sulfated GLP contains about 18.58% sulfate content. Sulfated GLP exhibited good antioxidant activity in terms of DPPH, hydroxyl, and alkyl radical-scavenging activity (0.10, 0.02, and 0.17 IC_50_, mg/mL, respectively). Studies showed that guava leaves extracts (GLE) effectively reduced the oxidative stress and toxicity caused by hydrogen peroxide in mammalian cell lines (Vero cells) [18]. GLPs are also found to be beneficial in treating diabetes mellitus symptoms. Acarbose (an antidiabetic drug) is commonly used for the treatment of type 2 diabetes [16]. It acts as an inhibitor of glycoside hydrolases like α-glucosidase and α-amylase and thus prevents rapid glucose release from complex carbohydrates [19]. This activity causes some of the incompletely digested complex carbohydrates to remain in the intestine and be transported to the colon. The intestinal microflora digests these complex carbohydrate fractions, causing gastrointestinal problems like diarrhea and flatulence. A study reported that GLP inhibited α-glucosidase more efficiently than acarbose without significantly blocking the α-amylase activity [19]. Moreover, it also caused a substantial drop in fasting blood sugar, total cholesterol, total triglycerides, glycated serum protein, creatinine, and malonaldehyde in diabetic mice without causing any major side effect [15]. Therefore, GLP can be used as a replacement of acarbose for managing diabetes mellitus and also as an antioxidant additive in foods.

**Table 1:** Nutritional profile of guava leaves.

Compounds	Content/Composition	References
Elements and ascorbic acid		[20]
Potassium	1.11%	
Phosphorus	0.23%	
Nitrogen	1.02%	
Ascorbic acid	142.55 mg/100 g	
Carbohydrates/phenols/sulfates		[18]
Fucose	1.44%	
Rhamnose	3.88%	
Arabinose	22.6%	
Galactose	29.41%	
Glucose	33.79%	
Mannose	0.59%	
Xylose	7.71%	
Phenol	15.28%	
Sulfate	18.58%	
Carbohydrate	48.13%	
Sulfate polysaccharide	66.71%	
Protein		
Association of Official Analytical Chemists (AOAC) method	22.98 ± 0.036% [dry weight (DW) basis]	[21]
AOAC method	9.73%	[22]
Lowry’s method	16.8 mg/100 g	[23]
Ninhydrin method	8.0 mg/100 g	

#### 2.1.2. Proteins

Guava leaves contains 9.73% protein on a dry weight basis [22]. Proteins are large biomolecules composed of amino acids and act as building blocks of cells. Proteins play a major role in growth and maintenance, enzyme regulation, and cell signaling, and also as biocatalysts [24]. Recently, plant-based nutrients have gained potential because of the high demand for nutritionally rich food, particularly protein. A great effort is now being made to find highly sustainable nutritionally rich food sources [25]. Thomas et al. [23] reported 16.8 mg protein/100g and 8 mg amino acids/100g in guava leaves as estimated according to Lowry’s and ninhydrin methods, respectively. Jassal et al. [21] reported that guava leaves can be utilized as a novel and sustainable dietary source as they are a rich source of proteins, carbohydrates, and dietary fibers.

#### 2.1.3. Minerals and Vitamins

Guava leaves are the rich source of minerals, such as calcium, potassium, sulfur, sodium, iron, boron, magnesium, manganese, and vitamins C and B. The higher concentrations of Mg, Na, S, Mn, and B in GLs makes them a highly suitable choice for human nutrition and also as an animal feed to prevent micronutrient deficiency [26]. Thomas et al. [23] reported the concentration of minerals such as Ca, P, K, Fe, and Mg as 1660, 360, 1602, 13.50, and 440 mg per 100g of guava leaf dry weight (DW), respectively. The concentration of vitamins C and B was 103.0 and 14.80 mg per 100g DW, respectively. Consumption of Ca- and P-rich GLs reduces the risk of deficiency-related diseases like hypocalcemia, hypophosphatemia, and osteoporosis. The study also reported that the concentration of Ca, P, Mg, Fe, and vitamin B in GLs was higher than that in guava fruit. The higher vitamin C content in GLs may help in improving the immune system and maintain the health of blood vessels, whereas vitamin B plays an important role in improving blood circulation, nerve relaxation, and cognitive function stimulation.

### 2.2. Phytochemical Profile

#### 2.2.1. Essential Oil Profile

GLs are a rich source of essential oils (Table 2). The major constituent of GL essential oil includes 1,8-cineole and *trans*-caryophyllene [27]. Chen et al. [8] identified 50 compounds in GL essential oil using gas chromatography (GC) and gas chromatography/mass spectrometry (GC–MS), where they found β-caryophyllene, α-pinene, and 1,8-cineole to be the major ones. GL essential oil from the Philippines was found to contain a different profile, with limonene, α-pinene, β-caryophyllene, and longicyclene as major compounds [28]. Ecuadorian GL essential oil contained a higher content of monoterpenes (limonene and α-pinene) whereas Tunisian guava leaf oil displayed a higher content of veridiflorol and *trans*-caryophyllene [29,30]. Soliman et al. [31] reported a larger amount of monoterpenes, contrary to the other studies, where sesquiterpenes constituted the major compound in GL essential oil. El-Ahmady et al. [32] reported 4 α-selin-7(11)-enol, α-selinene, β-caryophyllene, and β-caryophyllene oxide as the major constituents of GL essential oil. In another study, sixty-four different compounds were determined in essential oil extracted from GLs by gas chromatography–mass spectrometry (GC–MS). Among them, caryophyllene (24.97%) was found to be predominantly present, which acts as an antioxidant, anticancer, anti-inflammatory, and antimicrobial agent [21]. This study reported the concentration of non-oxygenated sesquiterpenes, oxygenated sesquiterpenes, and monoterpenes as 73.67, 12.94, and 8.55%, respectively.

#### 2.2.2. Phenolic Compounds

**Figure 2:**
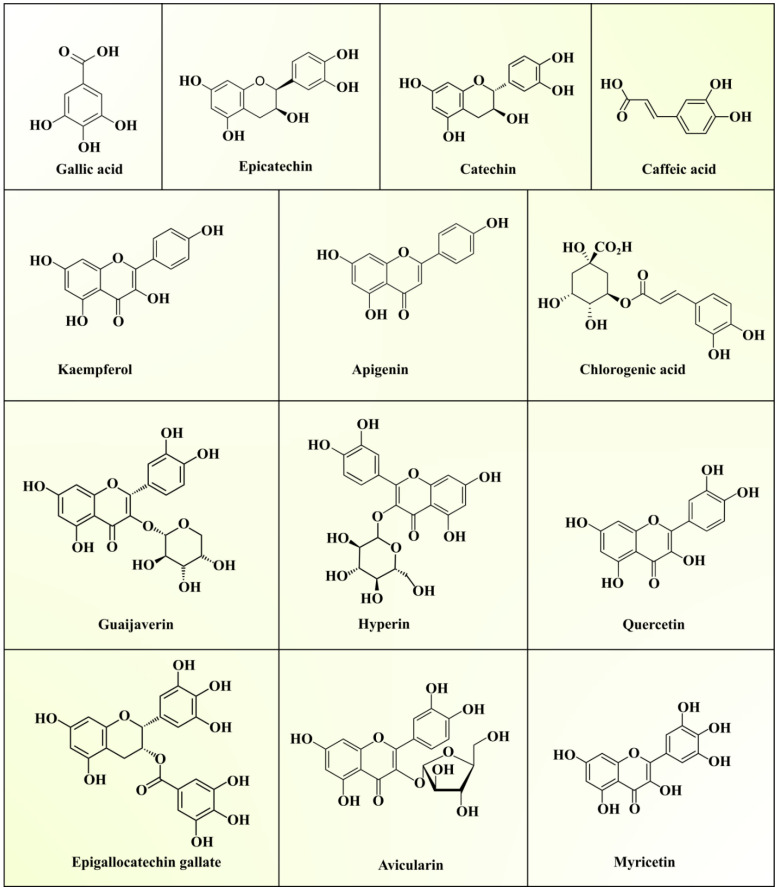
Structures of phenolic compounds present in guava leaf extracts.

GLs are widely popular as a traditional source of medicine in Asian countries due to their antihyperglycemic effect. As mentioned in the previous sections, they contain superior quality bioactive polysaccharides, proteins, lipids, essential oils, vitamins, and minerals. The various secondary metabolites present in GLs include phenolic acids, flavonoids, triterpenoids, sesquiterpenes, glycosides, alkaloids, and saponins. Phenolic compounds (PCs) serve as key bioactive compounds which provide antioxidant and hypoglycemic properties to GLs. Generally, these PCs play a major role in managing various metabolic and physiological activities in the human body. About seventy-two different phenolic compounds have been determined in GLs using high-performance liquid chromatography–diode array detector–quadrupole time-of-flight tandem mass spectrometry [33]. Generally, five quercetin glycosides are present in GLs. The presence of two new benzophenone galloyl glycosides (guavinosides A and B) and one quercetin galloyl glycoside (guavinoside C) was also reported [34]. Seventeen types of triterpenoids, thirty types of flavonoids, and nineteen types of sesquiterpenoids in GLs have also been reported [10]. Moreover, diphenylmethane [35] sesquiterpenoid-diphenylmethane meroterpenoids (psiguadials A and B) [36] and psiguanins A–D (1–4) [37] were also found in GLs. Epidemiological studies have established the roles of polyphenolic compounds against chronic diseases, such as diabetes, cancer, and neurodegenerative and cardiovascular diseases [38]. Phenolic compounds modulate numerous physiological processes like cell proliferation, enzymatic activity, cellular redox potential, and signal transduction pathways to fight against chronic pathologies [39]. Various phenolics reported in GLs are summarized in Table 3 and the structures can be seen in Figure 2.

Among phenolic compounds, quercetin is a major bioactive phenolic compound in GLs. Diets enriched with bioactive compounds have been gaining much attention in recent years due to their potential to lower the risk of the development of numerous chronic diseases. Seven pure compounds, quercetin, avicularin, apigenin, guaijaverin, kaempferol, hyperin, and myricetin, were separated from the ethyl acetate (EtOAc)-soluble GL fraction using Sephadex LH-20 column chromatography with reversed-phase thin layer chromatography (RP-TLC) to monitor separation. Mass spectrometry and nuclear magnetic resonance spectroscopy were used to elucidate the compound structures [40]. Wang et al. [43] extracted and analyzed phenolic compounds from non-fermented guava leaves (NFGLs) and fermented guava leaves (FGLs) using high-performance liquid chromatography coupled to electrospray ionization quadropole–time-of-flight mass spectrometry (HPLC–TOF–ESI/MS). The authors reported the presence of gallic acid, rutin, chlorogenic acid, avicularin, isoquercitrin, quercitrin, and kaempferol in NFGL and FGL samples. Among them, quercetin, rutin, gallic acid, avicularin, and isoquercitrin occupied about 65% of the total peak area on the chromatogram. Another study reported higher concentrations of catechin (2.25%) and epicatechin (1.45%), whereas gallic acid, chlorogenic acid, quercetin, caffeic acid, and epigallocatechin gallate were present in lower concentrations in GL extract [41]. Additionally, phenolic compounds (eugenol and isoeugenol) and alkaloids (cevadine and emetine) were detected. Díaz-de-Cerio et al. [44] optimized the extraction of proanthocyanidins, as antidiabetic and antiobesity agents [45], from GLs by HPLC–fluorimetric detector (FLD)–ESI–MS and studied their degree of polymerization in different oxidation states. Thus, the phytochemical profile of GL extract depicts the presence of numerous phytochemicals with distinct medicinal properties, suggesting its application to cure human diseases.

## 3. Biological Activities of Guava Leaf Extracts

The compounds from guava leaf extracts possess multidirectional biological activities, including antioxidant, hypoglycemic, anticancer, and other biological activities. It was also reported that polysaccharide fractions of sulfated GLP possess stronger biological activities, such as antioxidant, antibacterial, and antitumor effects compared to unsulfated ones. The useful bioactivities of GL extract are presented in the following subsections.

### 3.1. Anticancer/Antitumor Activity

Cancer is a complex health disorder which is identified by the development of cell proliferation or a decrease, causing apoptosis [46]. It can be caused by several exogenous and endogenous factors involved in the excessive production of reactive oxygen species (ROS). This can result in single- or double-strand breaks in DNA or RNA, base mutations, chromosomal breaking and reorganization, DNA cross-linkage, nucleic acid degradation, damage to cell membrane integrity due to lipid peroxidation, and tumor formation [47]. GLs are a good source of triterpenoids, sesquiterpenes, tannins, psiguadials, volatile oils, flavonoids, benzophenone glycosides, and miscellaneous quinones [10]. Psiguadial D and psiguadial C act as inhibitors of human hepatoma cells (HepG2) and protein tyrosine phosphatase 1B (PTP1B). Terpenoids and flavonoids present in GLs exhibit antitumor effects by regulating the immune system, suppression of signal transfer and tumor cell adhesion, and an impediment to tumor angiogenesis and cell proliferation [48]. Studies suggest that these leaves exhibit a potent inhibitory effect against cancer cell lines like MDA-MB-231 and Michigan Cancer Foundation-7 (MCF-7) for breast cancer, Henrietta Lacks (HeLa) for cervical cancer, KB for nasopharyngeal cancer, LNCaP, DU 145, and prostate cancer-3 (PC-3) for prostate cancer, and colorectal 320 double minutes (COLO320DM) for colon cancer [49].

The growth of colorectal tumors chiefly relies on angiogenesis, a process by which new blood vessels develop from pre-existing ones. Prolonged angiogenesis is vital for the progression of tumors towards malignancy since the blood vessels efficiently supply the developing tumor cells with vital metabolites and oxygen and it also functions as an efficient means for cellular waste disposal. A study was conducted to investigate the anticancer and antiangiogenic potential of GL extracts against angiogenesis-dependent colorectal cancer [50]. Guava leaf extracts rich in vitamin E, flavonoids (apigenin), and β-caryophyllene demonstrated strong antiproliferative activity against human colon carcinoma cell lines Caco-2, HT-29, and SW480. The antiangiogenic property of β-caryophyllene is attributable to its interaction with the transcription factor HIF-1α that regulates the biological pathways related to hypoxia, tumor metastasis, and tumor-mediated angiogenesis. HIF-1α also mediates the transcription of vascular endothelial growth factor (VEGF) in the presence of β-caryophyllene, explaining the antiangiogenic and anticolorectal cancer property of guava leaf extract.

A caryophyllene-based meroterpenoid called guajadial from GLs was studied for its antiproliferative and antiestrogenic activities against human breast cancer cell lines MCF-7 BUS and MCF-7 [51]. The authors suggested that guajadial exerts its anticancer activity by acting on estrogenic receptors, induction of apoptosis by blocking DNA synthesis, and inhibition of the cell cycle at the G1 phase [52]. A similar study indicated that three benzophenones, guavinoside B, guavinoside E, and 3,5-dihydroxy-2,4-dimethyl-1-*O*-(6′-*O*-galloyl-β-D-glucopyranosyl)-benzophenone, isolated from guava leaves inhibited the growth of HCT116 human colon cancer cells [53]. These compounds strongly induced cancer cell apoptosis and modulated the expression of key proteins like extracellular signal-related kinases (p-ERK1/2), p53, c-Jun NH_2_-terminal kinases (p-JNK), and cleaved caspases 8 and 9, which are involved in apoptotic signaling and cell proliferation. Another study indicated the inhibitor effect of guava leaf extracts on lung cancer genes, primarily involved in signaling pathways like PI3K-Akt [10]. The authors stated that daidzein, ursolic acid, apigenin, genistein, and quercetin in the leaf extract strongly inhibited cyclin-dependent kinase 2,6 (CDK2,6), vitamin D_3_ receptor (VDR), hepatocyte growth factor receptor (MET), epidermal growth factor receptor (EGFR), progesterone receptor (PGR), peroxisome proliferator-activated receptor gamma (PPARG) and interleukin-2 (IL-2) proteins and subsequently blocked tumor proliferation and migration, tumor angiogenesis, tumor adhesion, and degradation of the extracellular matrix.

### 3.2. Antidiabetic Activity

Diabetes is a major chronic disease and about 10% of the world’s population suffer from blood glucose metabolic disorder, mainly characterized by a hyperglycemic condition. This situation is either characterized by insufficient secretion of insulin from β-cells of pancreatic islets (type 1 diabetes) or the inability of cells to react in response to the secreted insulin (type 2 diabetes) [12,54]. The International Diabetes Federation (IDF) stated that 451 million people were affected by diabetes mellitus, resulting in 5 million deaths, in 2017 and the global prevalence of diabetes is projected to hit 693 million cases by 2045 [55]. The prolonged condition of hyperglycemia leads to increased production of ROS and dyslipidemia, causing severe cellular damage and complications [56].

GLs have been widely used as ethnomedicine for diabetes management [15]. Flavonoids and polysaccharides of GLs have been reported for their antidiabetic potential in several studies. Guaijaverin and avicularin flavonoids of GL extract were associated with significant improvement in the function of β-cells of pancreatic islets and hepatocyte morphology in diabetic mice [57]. Guaijaverin suppressed the activity of the blood glucose homeostasis enzyme dipeptidyl-peptidase IV [58], while avicularin inhibited intracellular lipid aggregation by impeding glucose uptake through GLUT-4 in vitro and revealed no distinct toxicity for 3T3-L1 adipose cells [59]. Luo et al. [14] extracted GL polysaccharides (GLPs) and further tested the antidiabetic effects on streptozotocin-induced diabetic mice in combination with a high-fat diet. The authors revealed that GLP was associated with a significant reduction in total cholesterol, triglycerides, glycated serum protein, creatinine, fasting blood glucose, and malonaldehyde content, and increased total superoxide dismutase and total antioxidant capacity enzyme activity in vivo. Suboptimal glycemic regulation may lead to elevated postprandial glucose concentrations. Nair et al. [60] suggested that the inhibitors of α-amylase and α-glucosidase enzyme can decline postprandial glucose absorption, and are therefore possible targets for diabetes management. The polysaccharides were isolated from GLs by ultrasound-assisted extraction and the antiglycation activity of extracted polysaccharides was studied [16]. The authors found that GLP showed strong inhibition of α-glucosidase, with a 99.54% inhibition rate at a 100 μg/mL concentration, and less inhibition of α-amylase, with a 14.06% inhibition rate at a 1mg/mL dose concentration. The findings suggests that bioactive compounds from GLs can be effective in reducing the risk of diabetes.

### 3.3. Antioxidant Activity

Oxygen is an important element for aerobes since it acts as a terminal electron acceptor during the respiration process, which is the key source of energy production. However, free radicals produced during metabolic processes are responsible for numerous ailments in the human body, namely, inflammatory diseases, ischemic diseases, neurological disorders, hemochromatosis, emphysema, acquired immunodeficiency syndrome, and many others [61]. The presence of phenolic compounds, such as gallic acid, pyrocatechol, taxifolin, ellagic acid, ferulic acid, and several others, is responsible for the antioxidant roles of GLs [8,13]. High-performance liquid chromatography analysis of GL extracts revealed the presence of seven major flavonoids: quercetin, hesperetin, kaempferol, quercitrin, rutin, catchin, and apigenin, while other bioactive compounds, such as kaempfertin, isoquinoline, and corilaginoline alkaloids, were also identified [62]. These compounds are the major compounds responsible for the antioxidant properties of GLs.

The significance of antioxidant compounds from GLs in minimizing the harmful effects of free radicals has been shown by numerous studies. Essential oils extracted from GLs were found to function as moderate antioxidants with an IC_50_ value of ~460.37 ± 1.33 μg/mL, as demonstrated by a DPPH assay [27]. The reduction of linoleic acid oxidation and the scavenging effect on peroxyl radicals were revealed by other such analyses on GL extract. The study also showed that there was a linear association between the antioxidant’s potency, the ability to scavenge free radicals, and the phenolic content of GL extract [8]. The protective effect of GL polysaccharide was studied in zebrafish. The authors revealed that GL polysaccharides exerted a protective effect against oxidative stress induced by hydrogen peroxide by inhibiting the formation of reactive oxygen species (ROS), reducing lipid peroxidation and cell death [18]. In another study, it was revealed that GL extracts at 4000 ppm or higher can prevent the oxidation of fresh pork sausages, suggesting its application as a functional food ingredient [63]. To release insoluble bound polyphenol components, GLs were co-fermented with yeast and bacterial strains and it was observed that fermentation enhanced the antioxidant ability of soluble guava leaf polyphenols [43]. In an advanced study, silver nanoparticles were synthesized by utilizing crude polysaccharides of GLs, and showed high DPPH radical- and ABTS radical cation-scavenging activity [64]. It is evident from the findings that GL extracts can be a useful antioxidant material in the food preservation and cosmetic industries.

### 3.4. Antidiarrhea Activity

Currently, diarrhea is one of the prominent root causes of mortality among children in the age group of 0–5 years. Attempts have been made to discover new drugs with minimal side effects on the other organs of the body. In developing nations, attention has been devoted to identifying novel phytochemicals derived from medicinal plants to develop new drugs with minimal side effects [65]. Most pharmaceutical industries are engaged in the innovation of different drugs which have therapeutic potential to combat this disease. A number of therapeutic treatments are available for treating diarrhea in the form of synthetic drugs which cause many side effects in the human body, such as constipation, intestinal obstruction, induction of bronchospasm, and vomiting [66,67]. To combat these side effects, focus should be directed to investigate and isolate potent bioactive compounds from medicinal plants. GLs are considered to possess antidiarrheal properties, as reported by many researchers. Mazumdar et al. [12] reported the antidiarrheal potential of ethanolic isolates of GLs in Wistar rats. The authors reported that a dosage level of extracts at a concentration of 750 and 500 mg/kg had antidiarrheal potential in castor oil-fed rats. Besides this, Ojewole et al. [68] reported similar activity using aqueous extracts of GLs in rodents. They reported that GL extracts at doses of 52–410 mg/kg when administrated orally were found to combat diarrhea, and also resulted in reduced intestinal transit and dilatory removal of unwanted gastric products. Further, loperamide (13 mg/kg, p.o.) reduced the occurrence of defecation, along with the severity of diarrhea in the same animal models. The GL extracts reduced diarrheal symptoms, such as secretion of interstitial fluid and wetness of fecal droppings in a dose-dependent manner. GLs at different concentrations in rabbits showed concentration-dependent pulsing and pendulum retrenchments in the duodenum. These studies suggested that GLs possess excellent potential to combat diarrhea and they have gained the focus of scientific communities regarding their pharmacological prospects in different communities. In another study, Dewi et al. [11] proved the antidiarrheal potency of GL water extracts. The authors used the combination of GL water extract (G) and green tea leaf extract (T). The extracts were used in different combinations, i.e., (G:T) 112.5:110.55, (G:T) 75:221.1, and (G:T) 37.5:331.65 mg/kg body weight. The outcomes proved that all combinations had potent antidiarrheal activities, depicted by enhanced stool weight, stool onset, stool consistency, and diarrhea period. When mice were administered with a water extract of (G:T) 75:221.1 at a time interval of 180–240 min, a significant reduction in diarrhea was observed. Based on the above observations, it can be concluded that GLs demonstrated potent antidiarrheal efficacy. It may interesting to investigate, in future, the associated underlying molecular mechanism/s and also long-term toxicity studies in different animal models, as well as in human patients, to ascertain the therapeutic efficacy and safety.

### 3.5. Antimicrobial Activity

The evolution of novel disease-causing strains and resistance of microbes to classical antibiotics are currently serious concerns. The incidence of systemic microbial infections such as septicemia, urinary tract infections, meningitis, pneumonia, and gastritis affects the entire human body and contributes significantly to global mortality. Food-borne diseases are mostly caused by pathogens including *Staphylococcus*, *Shigella*, *Salmonella*, *Bacillus*, *Escherichia coli*, *Clostridium*, and *Pseudomonas* [69]. Plant-derived bioactive compounds are promising sources of antimicrobials. These compounds act by the inhibition of microbial cell wall development, disruption, and lysis, hampering biofilm formation, repression of DNA replication and transcription, impeding adenosine triphosphate (ATP) production, suppression of bacterial toxins, and the generation of reactive oxygen species (ROS) [70]. GLs, owing to the presence of different organic and inorganic antioxidants and anti-inflammatory compounds, are known to possess antimicrobial properties [71]. GL essential oils display strong antimicrobial properties against *Pseudomonas aeruginosa*, *Escherichia coli*, *Streptococcus faecalis*, *Staphylococcus aureus*, and *Bacillus subtilis* [31]. Studies also indicate their antioxidant and antiproliferative activities.

**Figure 3:**
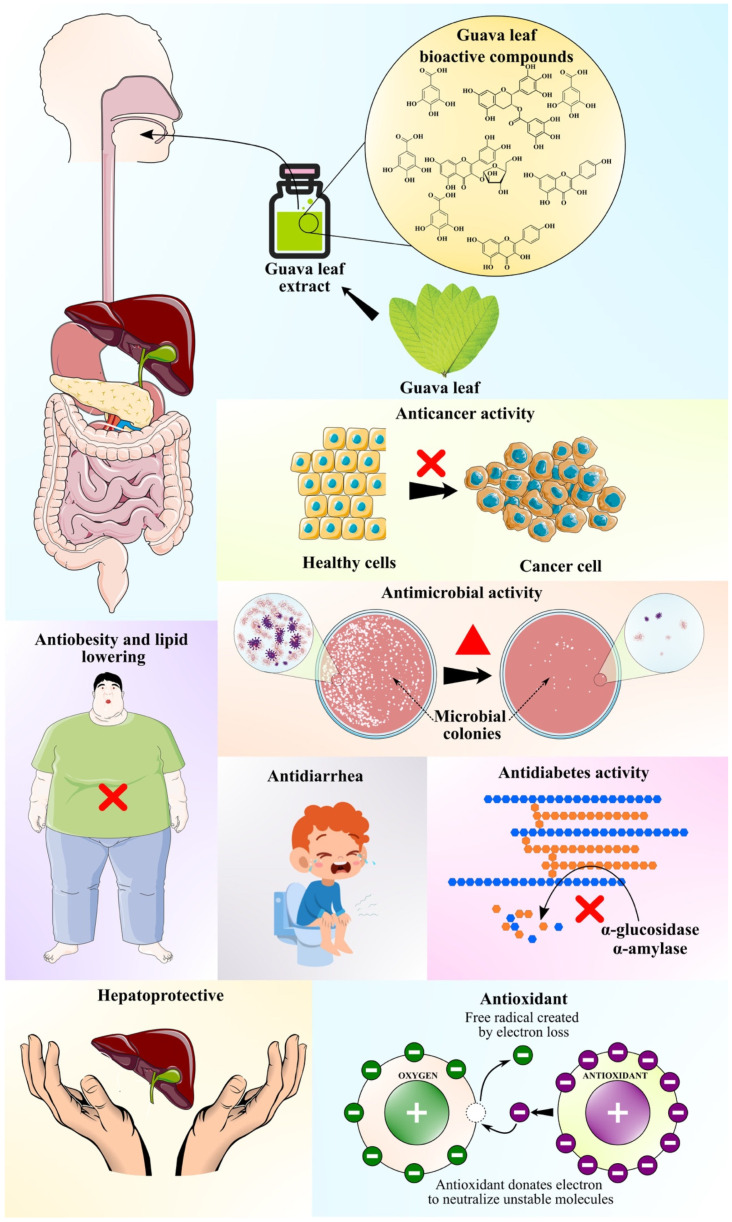
Various bioactivities of guava leaf extracts.

Qualitative analysis of aqueous and organic extracts of guava leaves revealed the presence of phenolic acids, flavonoids, terpenoids, glycosides, and saponins, in which their presence is positively correlated with antimicrobial activity. HPLC–TOF–ESI/MS analysis of fermented GLs confirmed the presence of gallic acid, chlorogenic acid, rutin, isoquercitrin, avicularin, quercitrin, kaempferol, morin, and quercetin. These compounds have the property of inhibiting ergosterol, which is a fungal cell membrane component, and glucosamine, which is a fungal cell growth indicator. Similarly, water-soluble tannins present in GLs act as bacteriostatic agents, with mechanisms of actions like withholding substratum, hampering oxidative phosphorylation, and extracellular enzyme inhibition. They have been demonstrated to have an inhibitory effect on antibiotic-resistant clinical isolates of *Staphylococcus aureus* [72]. Another study, by Hirudkar et al. [73], identified quercetin as one of the most predominant flavonoids of GLs with the highest pharmacological activity. Additionally, activity against bacterial and fungal pathogens was traced to triterpenoids like betulinic acid and lupeol [74]. A methanolic GL extract demonstrated antibacterial activity against *E. coli* with a minimum inhibitory concentration (MIC) of 0.79 μg/mL, a minimum bactericidal concentration of 51 μg/mL, and a reasonable antifungal activity with a minimum inhibitory concentration of 12.6 μg/mL [75]. A decoction of GLs at various concentrations (1%, 5%, and 10%) exhibited an inhibitory effect on bacterial colonization and binding of bacterial enterotoxins on epithelial cells, thus altering the inflammatory response. Guava extract showed higher activity of the antioxidant enzymes peroxidase, catalase, and polyphenol oxidase [76]. Biogenic production of silver nanoparticles (40 nm in size) using GL extract showed antibacterial activity against *Pseudomonas aeruginosa* owing to high antiradical activity against DPPH radicals and ABTS radical cations [77]. The role of the cytokine interleukin-7 (IL-7) as an immune booster against microbial infections is well studied. It is hypothesized that GL extracts act on the intestinal mucosal cells and aid the upregulation of IL-7 synthesis and help in the development of B and T cells [78]. Ongoing investigations on the antimicrobial activity of plant bioactive compounds encourage the utilization of GL extract in the treatment of microbial infections, oxidative stress-related diseases, and unraveling more prophylactic compounds from GLs. An overall presentation of all the bioactivities demonstrated by GLs is presented in Table 4 and Figure 3.

### 3.6. Hepatoprotective Properties

Liver lipid metabolism requires the activity of adenosine monophosphate-activated protein kinase (AMPK) and PPARα and rats treated with guava leaf extract demonstrated enhanced activity of both parameters. In addition, the guava leaf extracts could ameliorate hepatic insulin resistance. Alanine transaminase (ALT) and aspartate aminotransferase (AST) are associated with the functioning of the liver. Increases in their levels are an indication of fatty liver, which could be restricted with the administration of guava leaf extract [85]. Additionally, it has been found that diabetes has a close association with malfunctioning of the liver, including liver enlargement, steatosis, and fibrosis, as the primary function of the liver is to stabilize blood glucose levels. Any abnormality in the metabolism of glucose, lipid, and insulin is seen as a classic condition in type 2 diabetes mellitus. The bioactive compounds guaijaverin and avicularin present in guava leaves are potent inhibitors of dipeptidyl-peptidase IV and glucose transporter 4 (GLUT4)-mediated glucose uptake, respectively, responsible for raising the blood glucose levels [58,59]. Treatment with guava leaf extract with enhanced flavonoid levels promoted insulin resistance and restricted the rise in glucose as well as lipid levels in type 2 diabetes mellitus rats [57].

### 3.7. Antiobesity and Lipid-Lowering Activity

GLs are known to produce an antidiabetic effect and, therefore, are used for the treatment of diabetes [86]. Treating diabetic rats with 200 mg/kg body weight (b.w.) GLs caused a reduction in the blood glucose levels and promoted oral glucose tolerance, which is essential to prevent weight loss as a consequence of impaired carbohydrate metabolism. With the improved activity of hexokinase and G6PDH, and reduced activity of gluconeogenic enzymes and glucose-6-phosphatase, the insulin levels stabilized [87]. Similar findings were reported by [82].

Hypercholesterolemia, or high cholesterol levels in the blood, occurs as an effect of faulty dietary habits, genetics, or improper lifestyle. GLs contain many bioactive compounds with antioxidant properties which have health-promoting functions [88]. Wang et al. [40] reported the presence of flavonoids such as quercetin, kaempferol, guaijaverin, avicularin, myricetin, hyperin, and apigenin in guava leaf extract. Additionally, these flavonoids contributed to inhibitory action against α-glucosidase and α-amylase. It could be concluded from the study that the presence of the –OH group on the third position of the flavonoid was responsible for the inhibitory action. The inhibitory actions of myricetin, quercetin, and kaempferol against α-glucosidase and α-amylase were the highest, but a synergistic effect was quite evident. The presence of glycosides is essential to carry out the inhibitory function. Administration of ethanol extracts of guava leaves in the diet of rabbits brought about a significant reduction in the levels of serum triglycerides and low-density lipoprotein, with alleviated high-density lipoprotein levels [89]. Similar findings were reported in rats suffering from chronic diabetes along with hyperlipidemia [90].

## 4. GLs as a Functional Food Ingredient

Recent articles have shown that plant byproducts, such as fruit or vegetable pomace, seeds, husk/bran/seed coat, peel, and leaves, are important source of bioactive compounds and can be utilized as functional food ingredients [54,91,92,93,94,95,96,97,98,99]. Numerous reports suggest the beneficial effects of the inclusion of GL extract in food as a functional food ingredient, because of the presence of a myriad of compounds like rutin, naringenin, gallic acid, catechin, epicatechin, kaempferol, isoflavonoids, vitamins, citric acid, and flavonoids such as quercetin and guaijaverin, which are well known for their antimicrobial, antioxidant, and anti-inflammatory actions [100]. A study on the hypoglycemic effects of GL extract, due to the presence of its phenolic compounds, were shown to improve vascular dysfunction in mice with diet-induced obesity [101]. Recently, GL extract has been used in the preparation of jelly with pectin and was subjected to mass spectrometry analysis, which verified the presence of quercetin, gallocatechin, esculin, 3-sinapoylquinic acid, ellagic acid, gallic acid, and citric acid that are responsible for antioxidant and antimicrobial properties. Additionally, the addition of GL did not cause any change in the texture properties of the jelly [102]. The potentiality of GL as a functional immunostimulant ingredient in fortified foods, owing to the presence of a high level of antioxidant and phenolic compounds, was also studied in detail [7]. Another study on the evaluation of food–drug interactions of guava leaf tea (GLT), which is a functional food and beverage that is commercially available in Japan, showed no possibility of interactions between GLT and medicines, indicating the safety of GLT in terms of food–drug interactions. Borderline diabetics, who are at high risk of the development of diabetes, take GLT to suppress a rapid increase in blood sugar level after meals. GLT consists of carbohydrate and dietary polyphenols which bind to digestive enzymes and are known to contribute to health through poor absorption of dietary sugar or lipids [103]. Furthermore, a recent report that studied herbal tea also stated that guava tea showed no interaction with medicine [104]. Another study on the addition of yellow strawberry GLs with abundant phenolic and flavonoid compounds in the diet of laying hens showed antimicrobial and antioxidant effects which could enhance the quality of eggs through the mechanism of inhibiting the pathways of the enzyme cyclooxygenase (COX), which plays a fundamental role as an inflammatory mediator [105]. In addition, the natural antioxidants present in GLs after fortification in fresh pork sausage were found to be effective in decelerating the process of lipid oxidation in the fresh pork sausage [63]. These examples indicate that GL is an excellent source of active compounds for functional ingredient additives in foods, without altering the rheological and sensory properties.

## 5. Conclusions and Future Perspectives

GLs are documented as a source of natural compounds that are readily available. GL extracts have been extensively studied for their high levels of antioxidant, anticancer, hypoglycemic, and other biological activities. The rich presence of minerals and proteins, as well as vitamins, in GLs promote their utilization as a direct source of nutrients. The presence of numerous bioactive chemical compounds in GLs have been reported to enhance and stabilize different physiological and metabolic functions in the human body. GL also contains many secondary metabolites, such as flavonoids, triterpenoids, sesquiterpenes, glycosides, alkaloids, saponins, and other phenolic compounds. These compounds play a key role as immune stimulators and modulators of chronic diseases including diabetes, cancer, and gastrointestinal, neurodegenerative, and cardiovascular diseases. GL essential oil also has antioxidant, antimicrobial, and antiproliferative activity. GL extracts that contain high concentrations of vitamin E, flavone (apigenin), or β-caryophyllene show significant antiproliferative activity against colon carcinoma and various forms of human cancer. GL therefore has peculiar characteristics and pharmaceutical and medicinal profiles that promote diverse applications as an essential plant component in medicinal research and a low-cost ingredient in foodstuffs. Furthermore, as an ingredient of plant origin, GL may help in mitigate drug resistance, which is a major problem for the pharmaceutical industry and also can be utilized as a functional food ingredient, which are in high demand. Thus, guava extract with its multiple medicinal properties needs to be further developed for wider applicability. In future studies, the identification and isolation of new chemical components for the development of specific products will be the key research area.

## Figures and Tables

**Table 2:** Essential oil components of guava leaves.

Compounds	Content/Composition	References
Essential oil components		[31]
α-Pinene	1.53%	
Benzaldehyde	0.83%	
*p*-cymene	0.52%	
Limonene	54.7%	
1,8-Cineole	32.14%	
β-*cis*-Ocimene	0.28%	
γ-Terpinene	0.38%	
α-Terpineol	1.79%	
β-Caryophyllene	2.91%	
α-Humulene	0.77%	
Total identified constituents	95.85%	
Caryophyllene, copaene, nerolidol, caryophyllene oxide, humulene, limonene, eucalyptol, beta-bisabolene, cadin-4-en-10-ol, *trans*-cadina-1,4-diene, sesquiterpenes, eugenol, isoeugenol, cevadine, emetine (extracted from guava leaves, Ludhiana, India using hydro-distillation by Clevenger-type apparatus)	-	[21]

**Table 3:** Phenolic compounds of guava leaves.

Origin of Guava Leaves	Extract/Fraction	Bioactive Compounds	References
Leaves from Guangzhou (China)	Ethyl acetate-soluble fraction, n-butanol-soluble fraction, 75% ethanol extract, residual fraction, dichloromethane-soluble fraction	Quercetin, avicularin, apigenin, guaijaverin, kaempferol, hyperin, myricetin	[40]
Leaves from Jing-cin Farm (Tianzhong Township, Changhua County, Taiwan)	Aqueous extract	Gallic acid, catechin, epicatechin, quercetin, chlorogenic acid, epigallocatechin gallate, caffeic acid	[41]
Leaves from Motril (Spain)	Acetone, water, and acetic acid extract	Proanthocyanidins (PAs)	[33]
Leaves from Jiangmen (China)	Methanol extract	Gallic acid, chlorogenic acid, epicatechin, mono-3-hydroxyethyl-quercetin-glucuronide, rutin, isoquercitrin, quercetin-3-*O*-α-L-arabinofuranoside, quercetin-3-*O*-β-D-xylopyranoside, avicularin, quercitrin, kaempferol-3-arabofuranoside, quercetin, kaempferol	[42]

**Table 4:** Biological activities of guava leaf (GL) extracts.

Origin of Leaves	Type of Extracts	Bioactive Compounds	Type of Cell Lines, Type of Study	Results	References
Anticancer activity					
Leaves from Chaudhry Wala, Punjab, (Pakistan)	Extracts obtained using methanol, chloroform, and hexane	Phenolics including flavonoids	Human carcinoma cell lines (SCC4, U266, and KBM5)	IC_50_ values of the leaf extracts ranged from 22.73 to 51.65 mg/mL (KBM5); 20.97 to 89.55 mg/mL (U266); 22.82 to 70.25 mg/mL (SCC4). Hexane extract demonstrated strong cytotoxic (IC_50_ value = 32.18 μg/mL) and antitumor (IC_50_ value = 65.02 μg/mL) properties. These extracts also inhibited TNF-α and instigated NF-κB activation in KBM5 cells	[9]
-	Ethanolic extract	Chlorophyll	Glioblastoma cells (U-118 MG), colorectal adenocarcinoma cells (Caco-2), hepatocellular carcinoma cells (HepG2), breast cancer cells (MDA-MB-231 and MCF7)	IC_50_ values of the leaf extracts were >200 μg/mL for Caco-2, HepG2, MDA-MB-231, MCF7 and 133.55 for U-118 MG, demonstrating their potential	[79]
Leaves from Yaoundé (Cameroon)	Ethanolic extract and essential oils	β-Sesquiphellandrene, α-humulene, nerolidol, 1,8-cineole, isodaucene, benzaldehyde, *β*-bisabolol, β-caryophyllene	Hepatocellular carcinoma cells (HepG2) and healthy human skin fibroblasts (CCD-45-SK)	The IC_50_ values for aqueous and ethanol extracts of guava leaves against CCD-45-SK were >0.1 mg/mL and 0.1 mg/mL for essential oils. The IC_50_ values for aqueous ethanol extracts and essential oils against HepG2 were 0.013, 0.0057, and 0.1, respectively	[80]
Antidiabetic activity					
Leaves from Bangladesh	Ethanolic extract	-	Wistar rats with alloxan-induced diabetes	Administration of guava leaf extract significantly reduced (*p* < 0.05) BGL at doses of 1.00 and 0.50 g/kg, as well as 0.75 g/kg in alloxan-induced diabetic Wistar rats (*p* < 0.001)	[12]
Leaves from Guangdong (China)	Ultrasound-assisted ethanolic extract	Polysaccharides	In vitro	Inhibited α-glucosidase activity and reduced the breakdown of glucose and prevented flatulence by not attenuating α-amylase activity	[16]
-	65% ethanol and ethyl acetate extract	Flavonoids (guaijaverin and avicularin)	Kunming mice with high-fat diet and streptozotocin-induced diabetes	GLF (200 mg/kg/day) not able to prevent loss of body weight, which indicated the inability to remove the damage induced by streptozotocin Hypoglycemic effect, improved glucose tolerance. Decreased TC, TG, LDL-C. Improved the insulin resistance and function of beta cell islets. Reduced liver and kidney index. Reduced liver viscera index by reducing the accumulation of lipids in the liver	[57]
Leaves from Natal Province, (Republic of South Africa)	Lyophilized water extract	-	Sprague Dawley male rats with streptozotocin-induced diabetes	Guava leaf extract (400 mg/kg/d) significantly decreased HSL activity in diabetic rat liver and adipose tissue, which was associated with increased levels of glycogen, decreased total cholesterol, serum triglycerides, LDL-C, and increased HDL-C.	[81]
Leaves from Ambohitantely (Madagascar)		-	Rat hepatoma (H4IIE cells), adipocyte-like cells (3T3-L1), skeletal muscle cells (C2C12)	IC_50_ values of the leaf extract of 1.0 ± 0.3 inhibited α-glucosidase activity and significantly increased the accumulation of triglycerides in 3T3-L1 cells. Results demonstrated the application of guava leaf extract in the treatment of type 2 diabetes	[82]
Antioxidant activity					
-	Water extract	Low molecular weight polysaccharides (3.64 kDa)	In vitro antioxidant assays	IC_50_ values of 46.49 μg/mL, 175.52 μg/mL, and 102.82 μg/mL for DPPH, OH, and ABTS were recorded, respectively, all higher than that of ascorbic acid or Trolox	[15]
-	Water, methanol, and ethanol	Phenolics including flavonoids	In vitro DPPH assay	IC_50_ value was highest for ethanolic extract and the lowest for methanolic extract	[82]
Antidiarrheal activity					
-	Water extract	-	In vivo with rats and mice	PGE (50–400 mg/kg p.o.) produced dose-dependent and significant protection of rats and mice against castor oil-induced diarrhea, inhibited intestinal transit, and delayed gastric emptying	[68]
-	Ethanolic extract	-	In vivo Wistar rats	Application of EEPGL at doses of 750 and 500 mg/kg showed antidiarrheal effect in castor oil-induced diarrheal model	[12]
Antimicrobial activity					
-	Methanolic extract, water extract, extract of flavonoids	Alkaloids, saponins, anthraquinones, tannins, terpenes, flavonoids, coumarins	Antimicrobial activity of leaf extract was studied against *Bacillus subtilis*, *Staphylococcus aureus*, *Escherichia coli*, and *Salmonella typhi*	Methanolic extracts with minimum inhibitory concentration (MIC) of 5.5–11 mg/mL. Aqueous extract found to be least effective with MIC of 2–15 mg/mL. Flavonoid extract was the most effective against bacteria with MIC of 2.5–5 mg/mL	[83]
-	Ethanolic extract	-	Synergistic effect of zinc oxide nanoparticles and guava leaf extract for enhanced antimicrobial activity against enterotoxigenic *Escherichia coli*	Rifampicin (5 μg) zone of inhibition—28 mm Nanoparticle (concentration 128 μg/mL) zone of inhibition—24 mm Nanoparticle (concentration 128 μg/mL) + leaf extract zone of inhibition—20 mm	[84]

Abbreviations: KBM5—human chronic myelogenous leukemia; SCC4—human tongue squamous carcinoma cells; U266—human multiple myeloma cells; IC_50_—half maximal inhibitory concentration; TNF-α—tumor necrosis factor alpha; NF-κB—nuclear factor kappa light chain enhancer of activated B cells; Caco-2—cancer coli-2; MCF7—Michigan Cancer Foundation-7; GLF—guava leaf flavonoid, HSL—hormone-sensitive lipase; TC—total cholesterol; TG—triglycerides; BGL—blood glucose level; LDL-C—low-density lipoprotein cholesterol; HDL-C—high-density lipoprotein cholesterol.
